# A Short-Course Antibiotic Prophylaxis Is Associated with Limited Antibiotic Resistance Emergence in Post-Operative Infection of Pelvic Primary Bone Tumor Resection

**DOI:** 10.3390/antibiotics10070768

**Published:** 2021-06-24

**Authors:** Yoann Varenne, Stéphane Corvec, Anne-Gaëlle Leroy, David Boutoille, Mỹ-Vân Nguyễn, Sophie Touchais, Pascale Bémer, Antoine Hamel, Denis Waast, Christophe Nich, François Gouin, Vincent Crenn

**Affiliations:** 1 Orthopedics and Trauma Department, University Hospital Hotel-Dieu, UHC of Nantes, 44000 Nantes, France; yoann.varenne@chu-nantes.fr (Y.V.); myvan.nguyen@chu-nantes.fr (M.-V.N.); sophie.touchais@chu-nantes.fr (S.T.); denis.waast@chu-nantes.fr (D.W.); christophe.nich@chu-nantes.fr (C.N.); Francois.GOUIN@lyon.unicancer.fr (F.G.); 2 Bacteriology Department, University Hospital Hotel-Dieu, UHC of Nantes, 44000 Nantes, France; stephane.corvec@chu-nantes.fr (S.C.); Anne-Gaelle.leroy@chu-nantes.fr (A.-G.L.); pascale.bemer@chu-nantes.fr (P.B.); 3 CRCINA, INSERM, University of Angers, University of Nantes, 44000 Nantes, France; 4 Laboratoire EA 3826 “Thérapeutiques Cliniques et Expérimentales des Infections”, IRS2-Nantes Biotech, University of Nantes, 44000 Nantes, France; 5 Department of Infectious Diseases, CIC UIC 1413 INSERM, University Hospital, 44000 Nantes, France; david.boutoille@chu-nantes.fr; 6 Pediatric Orthopedic Surgery Department, University Hospital, UHC of Nantes, 44903 Nantes, France; antoine.hamel@chu-nantes.fr; 7 Anatomy Department, Medical Faculty, 44000 Nantes, France; 8 PhyOs 1238, INSERM, University of Nantes, 44000 Nantes, France; 9 Département de Chirurgie, Centre de Lutte Contre le Cancer Léon Bérard, 69008 Lyon, France

**Keywords:** pelvic tumor, sarcoma, surgical site infection, prophylactic antibiotic treatment, antibiotic resistance

## Abstract

Resections of primary pelvic bone tumors are frequently complicated by surgical site infections (SSIs), thereby impairing the functional prognosis of patients, especially in case of implant removal. Although prophylactic antibiotics play an essential role in preventing SSIs, there are presently no recommendations that support their appropriate use. This study aimed to assess the impact of a 24 h prophylactic protocol on the bacterial ecology, the resistance pattern, and the SSI healing rate. We hypothesized that this protocol not only limits the emergence of resistance but also results in a good cure rate with implant retention in case of SSI. A retrospective study was performed that included all patients with an SSI following a pelvic bone tumoral resection between 2005 and 2017 who received a 24 h antibiotic prophylaxis protocol. Twenty-nine patients with an SSI were included. We observed a 75.9% rate of polymicrobial infection, with a high prevalence of digestive flora microorganisms and a majority of wild-type phenotypes. We confirmed that there was no significant emergence of resistant flora. After first-line debridement, antibiotics (DA) if any implant was used, or debridement, antibiotics, and implant retention (DAIR) whenever possible, we obtained a 79.3% cure rate, with implant removal in 20% of cases. The absence of an implant was significantly associated with SSI healing. Early infection management and low resistance profiles may also have a positive effect, but this needs to be confirmed in a larger cohort. In light of this, the use of a 24 h prophylactic protocol in primary pelvic bone tumor resections is associated with a favorable infection cure rate and implant retention in case of SSI, and minimal selection of resistant microorganisms.

## 1. Introduction

Primary tumors of the pelvis are rare, accounting for no more than 15% of all primary bone tumors [1]. Yet, their surgical treatment generally requires a large resection, often impairing the patient’s functional prognosis. The challenge lies not only in the osseous reconstruction, but also in preservation of key elements as well as the urinary and intestinal tracts. Despite the expected surgical difficulties with obtaining healing via healthy margin resections, limb-salvage surgery has replaced amputations and has become the gold standard for surgical management of primary pelvic tumors, with or without prosthetic reconstruction [2]. However, the complication rate is high with this demanding surgery, and surgical site infections (SSIs) are a common occurrence [3,4]. The related large dead spaces and the proximity of the digestive tract result in a significant risk of SSI [4]. Although muscular flap coverage and the limitation of prosthetic material in tumor resection can reduce the incidence of infections [5,6,7], it seems self-evident that antibiotic prophylaxis must play a crucial role in reducing SSI, management of the bacteriological flora, and the emergence of antibiotic resistance.

The SSI incidence associated with bone tumor surgery ranges from 10 to 67% (depending on the series and the methodological criteria) for iliac bone and sacral tumor resection [4,8,9,10,11,12,13,14], compared to 1% in prosthetic conventional surgery. In this context, SSI can delay adjuvant chemotherapy or radiotherapy, thereby compromising the oncological prognosis. Moreover, their treatment is challenging, with high failure rates [3,4], and it involves surgical debridement and prolonged antibiotic treatment, and it sometimes requires removal of the implants when an acetabular reconstruction or massive prosthesis has been used [4,9]. This serious complication can significantly impact the morbidity and the functional outcomes after pelvic bone resection, with low healing rates resulting in a need for debridement, the administration of antibiotics, and implant retention (DAIR).

In conventional orthopedic surgery, first-generation cephalosporins (1GC) are commonly used for prophylaxis. Their narrow spectrum targets methicillin-susceptible Gram-positive cocci (GPC), which are often implicated in SSIs, as well as *Enterobacteriales*. Their use in a 24 h prophylaxis does not appear to promote the emergence of resistance [15]. Moreover, it was shown a few decades ago that infection rates are markedly diminished with a 24 h protocol compared to a prolonged protocol in surgical procedures, especially due to ecologic considerations [16]. In comparison, little is known about how to manage prophylaxis in pelvic resections, whether or not associated with a reconstruction. In contrast to common prosthetic infections, which are related to a clear predominance of GPC, pelvic SSIs are often caused by a polymicrobial population, with a strong representation of Gram-negative bacilli (GNB) and, generally, *Enterobacteriales* associated with anaerobic bacteria from the digestive flora [4,17]. Therefore, it might be appropriate to use a 1GC prophylaxis combined with a nitroimidazole drug [18], as suggested by anesthesia societies [19]. However, no clear international recommendations regarding either the type of drug or the treatment duration per- and post-operatively have been established to date. In this context, several teams performed a long-course antibiotic regimen for five days or more with pelvic resection surgeries to avoid this dreaded complication [3,20]. This approach raises the question of the emergence of resistance, specific microbiological profiles, and potential healing difficulties. In our center, we used a short 24 h antibiotic protocol in order not to underestimate potential early infection and biofilm issues, while also limiting microbiological selection pressure, as we hypothesize it may improve SSI management combined with a DA or DAIR procedure.

In light of the wide disparity in the duration of antibiotic prophylaxis protocols, along with the lack of recommendations, our monocentric study in a cohort of infected pelvic tumor resections aimed to describe the influence of a 24 h prophylaxis protocol. Therefore, an in-depth analysis of the bacteriological flora associated with SSI after pelvic tumor bone resection was carried out. Secondly, the antibiotic susceptibility was assessed in order to determine the associated risk of the emergence of resistance. Finally, we evaluated the impact of the chosen prophylaxis on SSI healing and implant retention, analyzing the healing rate after a first-line DA or DAIR procedure, and factors associated with healing and implant retention.

## 2. Materials and Methods

### 2.1. Study Design

We carried out a retrospective, monocentric, continuous study that comprised all of the patients treated between 2005 and 2017 in the orthopedic surgery department at Nantes University Hospital after a malignant pelvic bone tumor resection. All of the patients who met the Musculoskeletal Infection Society (MSIS) criteria in the first year following surgery were included and were considered to be infected with an SSI [21].

#### 2.1.1. Participants

The patients had to be over 16 years of age and operated for a curative procedure for a primary malignant bone affecting the iliac bone or the sacrum. The exclusion criteria comprised soft tissue sarcomas, a proximal femur tumor without acetabular involvement, tumors of the lumbar spine, and revision surgeries for pelvic tumors considered to be local recurrences. Of the 114 patients who met the inclusion criteria, 22 cases involving bone metastasis, 6 involving a hematologic malignancy, and 31 involving non-curative surgeries were excluded. For the remaining surgical cohort, the 29 patients considered to be infected one year postoperatively based on the MSIS criteria constituted our cohort of interest.

#### 2.1.2. Antibiotic Prophylaxis and Preventive Strategy

All of the infected patients received only 24 h of antibiotic prophylaxis, dispensed in the hour before the incision, so as to be as effective as possible [22]. From 2005 to 2012, patients received a 24 h single antibiotic prophylaxis comprising 1GC (cefazoline 2 g IV). Since 2012, they received a dual antibiotic prophylaxis combining 1GC with a nitroimidazole drug (metronidazole 1 g IV). After the initial dose, reinjections were performed at half the dose, for 24 h, every two antibiotic half-lives. If the surgery took more than 4 h, 1 g of cefazoline was reinjected once at H4, and then 1 g was administrated every 8 h (along with metronidazole 500 mg, since 2012). If the patient’s weight was more than 100 kg and their BMI >35 kg/m^2^, the dose was doubled in order to attain a sufficiently bactericidal concentration at the surgical site [23]. In case of allergies, we used clindamycin, vancomycin, or cotrimoxazole.

Before a pelvic bone tumor resection, a preoperative skin preparation was performed using povidone–iodine liquid soap, and no specific bowel preparation was done.

### 2.2. SSI Management

After the surgery, a systematic clinical and radiological follow-up was scheduled. Whenever an SSI was suspected, a DAIR (in case of material implantation) or DA (in case of no material implantation) procedure was performed as a first-line treatment, which could be repeated due to the complexity of pelvic reconstruction as a multiple debridement strategy [24,25]. It is usually recommended in the first month following surgery, but this delay is longer in complex oncologic situations such as pelvic tumors. Our center generally irrigates the surgical site with at least 3 L of saline solution under pressure, replacing the prosthetic mobile parts whenever possible. Patients with at least one intraoperative positive bacteriological sample during revision surgery within the first year after the resection surgery were considered to be infected regardless of material implantation or the depth of the infection. Patients were considered cured of the infection after their treatment when they had no active infection, fistula, or suppressive antibiotic therapy at the last follow-up. Implant removal was performed in case of DAIR strategy failure.

### 2.3. Microbiological Analysis

When an SSI was suspected, as mentioned above, a revision surgery was performed. At least four perioperative specimens (one liquid, three tissue specimens) from the operating site were collected in sterile vials. After the addition of sterile steel beads, the samples were crushed with a Retsch MM401 bead mill, as previously described [26]. Blood agar and chocolate agar plates were inoculated and incubated under a CO_2_-enriched atmosphere for seven days. Schaedler broths were also inoculated and incubated for 14 days. As they became cloudy and after at least five days of incubation, the broths were subcultured on Schaedler blood agar plates under an anaerobic atmosphere. The isolated bacteria were identified using VitekMS matrix-assisted laser desorption ionization-time-of-flight (MALDI-TOF) mass spectrometry analysis. Antibiotic susceptibility tests were performed according to the EUCAST guidelines.

### 2.4. Statistical Analysis

The patient data were collected retrospectively. We noted the demographic, tumor resection surgery, and the SSI management parameters. The qualitative and ordinal variables were compared by a Fisher’s exact test with the odds ratio (OR) and its 95% confidence interval (95% CI). The quantitative variables were analyzed with a nonparametric Wilcoxon–Mann–Whitney test; the qualitative variables were analyzed with a Chi^2^ or a Fisher’s test. Univariate and multivariate regression analyses (selecting variables with *p* < 0.15) were used to identify factors associated with infection healing. The alpha risk of all these tests was set at 5%, with a level of significance of *p* < 0.05. The data collection was performed using Microsoft^®^ Excel, and the statistical analyses were performed using IBM^®^ SPSS Statistics V25 software.

### 2.5. Ethics

The institutional review board approved the protocol. According to French legislation regarding anonymized data analyzed retrospectively (articles L.1121-1 paragraph 1 and R1121-2, Public Health Code), Nantes University Hospital has confirmed that approval from the ethics committee was not needed due to the non-interventional nature of the study, and thus no ethics committee approval was necessary at the time of the beginning of the study. The requisite processes were undertaken with the “Direction de la Recherche Clinique” (DRC) of the University Hospital of Nantes, France, and the “Commission Nationale de l’Informatique et des Libertés” (CNIL). The database was anonymized, and all the patients provided their verbal consent and received a form with information regarding the study.

## 3. Results

### 3.1. Study Population

Twenty-nine patients experienced an SSI within the first year following pelvic tumor surgery, and they were considered to be infected according to the defined criteria (Table 1 and Table 2).

The mean follow-up was 56 (±38) months. Most of the infected cases were male patients (75.9%) and more than half of our SSI cases involved chondrosarcomas (51.7%). The mean BMI of our patients was 26.8 (±4.8) kg/m^²^. Twenty patients were operated on for an iliac tumor and nine for a sacral tumor. Regarding the 20 iliac resections, we observed a zone 1 involvement in seven cases (35%), a zone 2 involvement in 15 cases (75%), and a zone 3 involvement in 14 cases (70%). Two Enneking regions were involved in 11 cases (55%) and the three regions were involved in four cases (20%).

The mean surgery duration was 479 (±172) min, with the use of material implantation in 51.7% of the surgeries. The remaining cases did not involve a prosthetic reconstruction. The perioperative mean blood loss volume was 2690 (±1786) mL. The combined visceral surgeries were performed at D-1 by coelioscopy in seven cases (70.0%) for visceral preoperative liberation, one Bricker associated surgery was performed at the same time, and one colostomy. The remaining three cases (30.0%) were perioperative rectal liberations.

### 3.2. Bacteriological Flora in SSI after Pelvic Tumor Bone Resection

For the 29 SSIs included, seven infections (34.1%) were monomicrobial and 22 (75.9%) polymicrobial (Table 3). A GNB was isolated in 21 patients (72.4%), of which 12 were *E. coli* (41.4%). *Enterococcus faecalis* was isolated in 15 patients (51.7%). Ten anaerobes (11.8%) were isolated in five patients. Of the seven monomicrobial infections, four were due to a GPC (two *Staphylococcus aureus*, one *S. lugdunensis*, and one *S. epidermidis*), three to a GNB (one *Proteus mirabilis*, one *E. coli*, and one *Enterobacter cloacae*). No specific clinical features were identified for these seven patients.

GNB, *Enterococcus* sp., and anaerobes (except *C. acnes)* were categorized into a digestive flora group. The other GPC and *C. acnes* were categorized into the skin flora group. *S. aureus* was analyzed separately. Finally, one patient was infected with *P. aeruginosa* associated with a cutaneous flora, and another patient with *Streptococcus mitis* group associated with a digestive flora. Microorganisms from digestive flora were the most represented in our ecology.

### 3.3. Antibiotic Resistance Analysis

**Figure 1:**
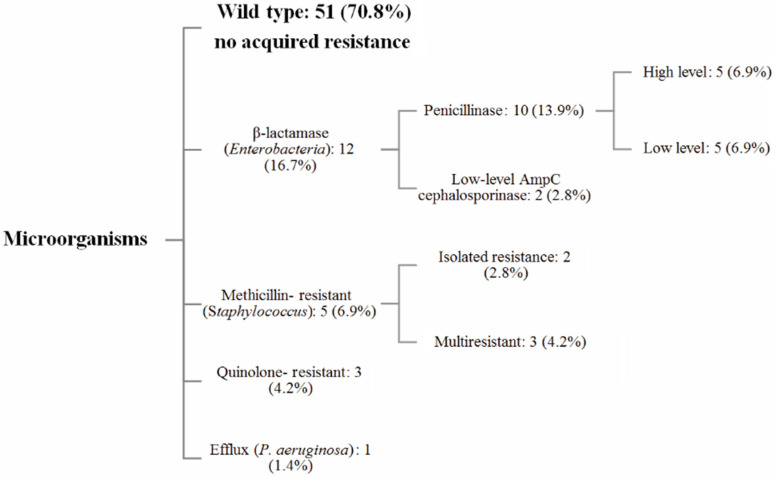
Representation of the resistance phenotypes of the microorganisms involved in the SSIs.

We analyzed the antibiotic susceptibility profiles of the bacteria involved in the SSIs (Figure 1).

Focusing on the resistance phenotypes of the 72 microorganisms identified, 51 strains (70.8%) exhibited a wild-type phenotype, while the other 21 microorganisms (29.2%) had acquired resistance. Five *Enterobacteriales* produced a low level of penicillinase, five produced a high level of penicillinase, and only two *E. coli* expressed a low level of cephalosporinase activity. Three anaerobes were resistant to fluoroquinolones. Five methicillin-resistant *S. epidermidis* (MRSE) were identified, including two multidrug-resistant MRSE (5 antibiotic families). One *P. aeruginosa* strain overexpressed the MexAB-OprM efflux pump. Interestingly, we did not find any evidence of *Enterobacteriales* producing extended-spectrum beta-lactamase (ESBLE) or overproducing their chromosomal cephalosporinase or harboring an acquired-carbapenemase. No methicillin-resistant *Staphylococcus aureus* (MRSA) were reported.

Irrespective of the antibiotic combination used (dual therapy or monotherapy), the SSIs involving anaerobic flora were not significantly different. The association of a nitroimidazole drug with 1CG lead to 25.0% anaerobic bacteria (two out of eight) vs. 14.3% (three out of 21) in the non-nitroimidazole group (1GC or co-trimoxazole or vancomycin), *p* = 0.620 (Table 4).

More microorganisms with acquired resistance were observed in patients with material removal or who had not healed from infection: 77.8% (7 out of 9), compared to the healed patients without material removal: 50.0% (10 out of 20), albeit not significantly (*p* = 0.234). Except for one isolated wild-type *E. Cloacae,* healing failures (i.e., fistula or suppressive therapy) were systematically associated with microorganisms that had acquired resistance: 83.3% (five out of six). The salvage external hemipelvectomy that was performed was infected by *E. faecalis* and a multidrug-resistant *S. epidermidis*. We also observed more healed patients with implant retention having an early infection occurring before one month, 75.0% (15 patients out of 20) than a late infection: 55.6% (5 out of 9), albeit not significantly (*p* = 0.396).

### 3.4. Patient Management and Healing Rate after a DA or DAIR Procedure

**Figure 2:**
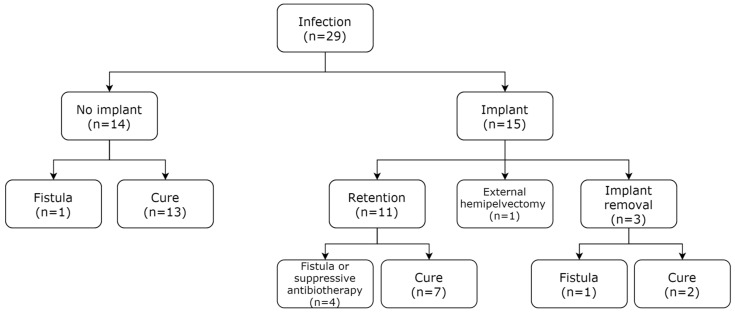
Outcomes for 29 patients with a pelvic tumor resection affected by infection and treated initially by debridement and antibiotics (DA) or debridement, antibiotics, and implant retention (DAIR).

With a first-line debridement, antibiotics procedure (DA) in no implant cases, or a DAIR strategy for each suspected SSI, we observed a cure rate of 79.3% (23 patients) at the last follow-up (i.e., no active infection, fistula, or suppressive therapy). After a DAIR procedure as a first-line treatment (*n* = 15), the implant was ultimately removed for three patients (20%) and external hemipelvectomy was necessary as a final treatment (*n* = 1). The three cases (20%) of implant removal after up to three DAIR surgeries underwent Girdlestone procedures, one with an active fistula, while the other two were cured of the infection (Figure 2). Regarding the DAIR procedures, with the objective of implant retention, antibiotic treatment was performed for three months and consisted of a dual antibiotic therapy in 86.7% of cases. The most widely used targeted antibiotic for DAIR management was quinolone, which was used in 80.0% of cases (Supplementary Table S1).

Multivariate analysis of the infection healing probability (excluding implant removal or hemipelvectomy) involved a three-variable model after univariate analysis, comprising the occurrence of early infection (<1 month) (*p* = 0.120), material implantation (*p* = 0.007), and the number of resistant bacterial entities (*p* = 0.119) (Table 5). In this analysis, material implantation was the most significant variable in the multivariate model (*p* = 0.023). The multivariate logistic regression model revealed a 79.3% overall predictive value.

## 4. Discussion

As SSI after pelvic resection is the predominant cause of reconstruction failure and morbidity during the first year, it is one of the most dreaded complications in tumor surgery, especially after reconstruction. Due to their high rate of occurrence, SSIs are a major concern for both the surgeon and the infection prevention team. Several prophylaxis protocols have been used to date, as there are no international recommendations in tumoral pelvic resection. Differences between drugs and the duration (from one to more than five days) of each regimen have been noted [3,18,27], and no studies to date have been focused on a short 24 h protocol in this situation.

With the aim of preserving the patient’s functional prognosis, by promoting a DA or DAIR procedure whenever possible, we suggest use of a short 24 h prophylaxis protocol, taking into account the specific multi-bacteriological flora involving GNB noted in these SSI, while bearing in mind the SSI ecology and avoiding raising the resistance rate.

### 4.1. Polymicrobial Infections in Pelvic Bone Resections

We observed a 75.9% incidence of polymicrobial infections, mainly from the digestive flora, often involving *E. faecalis* and particularly *E. coli* (a GNB), which was isolated in 72.4% of cases. These results are in line with previous findings: Abdul-Jabbar et al. described the influence of the anatomical level in spine surgery [28]: sacral surgery was significantly associated with an increase in polymicrobial infections (49.5%) and an increased number of infections by *Enterobacteriales*. Similarly, Vos et al. and Sanders et al. have also observed a high incidence of polymicrobial infections in pelvic bone resection, with Gram-negative bacteria as a particular risk factor for infection [3,13]. Ultimately, the causative microorganisms observed differ drastically from conventional periprosthetic joint infections, in which monobacterial Gram-positive infections predominate [29]. Finally, incision localization close to the groin has been reported to be a potential cause for this specific flora, while bacterial translocation through the intestinal wall is also a possible explanation, described as the “leaky gut” theory [3,30]. In light of these results, other prophylactic protocols could be considered, such as negative-pressure wound therapy, which is widely used in radiation therapy situations [31]. A selective digestive tract decontamination protocol using a per os combination of antibiotics has also been described and may be useful in pelvic sarcoma resection [32]. However, further assessments of these techniques are needed in order to evaluate the effects on bacteriological flora presentation.

### 4.2. Impact of Antibiotic Prophylaxis on the SSI Ecology: Avoiding Selection Pressure

In this surgical situation, the duration of antibiotic prophylaxis, as well as the choice of drugs, remain a matter of debate. The antibiotics chosen for prophylaxis have to be (i) tailored to the local ecology, (ii) not commonly used for antibiotic treatment, (iii) have a narrow spectrum of activity to avoid promoting resistance, and (iv) exhibit good diffusion and tolerance profiles.

#### 4.2.1. Prophylaxis Duration

As pointed out, there is currently no consensus in pelvic sarcoma surgery regarding the duration or the type of antibiotic prophylaxis. Yet in the review by Racano et al., the SSI rate appears to decrease with a protocol duration of more than 48 h [33]. Not surprisingly, extension of antibiotic therapy from between five to ten days is a widespread practice in numerous oncology surgical facilities [18,20].

However, this approach is double-edged, reducing the overall SSI rate on the one hand [8,33], while possibly compromising the infection management on the other hand. Indeed, a five-day prophylaxis may expose to a risk of a late SSI occurrence (>1 month), particularly in reconstruction procedures. This could lead to a longer bacterial biofilm settlement, and thereafter a decreased probability of material retention as the DAIR efficiency strategy declines [24,26]. Indeed, three weeks after the occurrence of SSI, a number of pathogenic microorganisms can form a membranous structure on the implant’s surface and enter the maturation stage. At this point, an open debridement is less likely to be able to completely remove the bacteria and their associated biofilm. Regarding this biofilm GPC issue, the efficacy of rifampicin is still a matter of debate, given the risk of the emergence of resistance. No data or recommendations for its use have been established to date in sarcoma pelvic resection [34].

#### 4.2.2. Choice of Prophylaxis Drugs

The bacteria that are usually targeted by prophylactic antibiotics in orthopedic surgery are the following: *S. aureus, S. epidermidis, C. acnes, Streptococcus* spp., *E. coli, and K. pneumoniae.* Therefore, the use of first-generation cephalosporins (1GC) such as cefazolin is recommended in conventional orthopedic surgery [35]. The multicenter study by *Sanders* et al. reported a variety of prophylaxis regimens: not only were 1CGs used, but also second-generation cephalosporins (2GC). Even combined prophylaxis such as clindamycin plus a third-generation cephalosporin (ceftriaxone) has been described [18]. Moreover, a nitroimidazole drug (metronidazole) or an aminoglycoside such as gentamicin have been added, depending on the surgeon’s preference and variables such as the duration of the surgery, the extent of resection, and the patient’s condition. The duration of these regimen was heterogeneous, ranging from one to five days [3].

However, as already mentioned, pelvic tumor resection surgery is mostly associated with GNB and anaerobic bacteria. Since 2005, we have, therefore, followed the national orthopedic surgery recommendations by using a short 24-h 1CG. Furthermore, in keeping with the colorectal surgery recommendations for pelvic surgeries, since 2012, we have added metronidazole [36]. However, the addition of a nitroimidazole entity as a short-term antibiotic prophylaxis in dual therapy does not appear to modify the ecology in terms of the occurrence of anaerobic microorganisms. Nonetheless, using a double prophylaxis, e.g., cefazoline plus metronidazole could be a consideration, as our results tend to indicate a higher SSI healing rate with this regimen used initially compared to 1GC only (albeit without significance, *p* = 0.160, in a univariate model). This effect still needs to be confirmed, however, with a larger population.

In addition, only a few publications to date have reported systematic data regarding prophylaxis in tumoral pelvic resection SSI outcomes. This, and the lack of protocol homogeneity, make it difficult to explore and compare the effectiveness of prophylactic antibiotics [3]. Efforts have nonetheless been made in the past decade using computational health engineering to model antimicrobial-pathogen interactions [37]. Through big data analysis and machine learning, Nocedo-Mena et al. have considered both drug and metabolic reaction networks at the same time in order to predict their activity on pathogenic microorganisms [38]. Other mathematical models based on time series have been developed, showing the quantitative effect of total antibiotic consumption on the level of bacterial resistance within a specific population [39]. These approaches could represent breakthroughs in the understanding of the complex subject of pelvic sarcoma SSI, although there is a need for more data in a multicenter context.

#### 4.2.3. Resistance Pattern Analysis

In terms of resistance patterns, most of the microorganisms involved in the SSIs in this study were susceptible to conventional antibiotics for bone and joint infections [40]. For the remaining microorganisms, we found that only 2.8% were MRSE and 3.2% were multidrug-resistant MRSE, for which conventional per os therapeutic options can be used such as cotrimoxazole, linezolid, and fusidic acid. Only one *P. aeruginosa* strain with an efflux-pump phenotype was identified in our cohort, and healing was managed in this situation. This resistance rate is low compared to the data in the literature, such as in Sciubba et al., who reported six cases (13%) of *P. aeruginosa* in a cohort of 46 patients operated for sacrum tumors [41]. Unfortunately, no data regarding prophylaxis have been reported in this series.

In the context of prosthetic material infection after tumor resection, using five days of 2CG prophylaxis, Dhanoa et al. noted an overall multidrug-resistance rate of 52.6%, which is almost twice the rate of 29.2% found in our cohort [27]. Moreover, they described worrisome increasing resistance rates from 2002 to 2011: methicillin-resistance (MRSE) increased from 30.4 to 43.9%, with the same trend for levofloxacin and teicoplanin resistance. We observed five MRSE (7.0%), but no MRSA were noted in our cohort. In addition, we did not find any ESBL producers, neither overproducers of chromosomal cephalosporinase or of carbapenemase. This is of particular importance, as these bacteria are currently undergoing significant development, particularly in Eastern Europe, and could lead to therapeutic dead ends [42,43,44]. Such resistance patterns have not been encountered in the flora described. Furthermore, the resistance pattern does not appear to play a role in our multivariate analysis model of the probability of infection healing. Overall, our short antibiotic prophylaxis approach may limit the emergence of ESBL producers. Furthermore, it does not appear to lead to an ecological selection pressure, which is a crucial point.

### 4.3. SSI Management: Healing and Implant Retention

#### 4.3.1. SSI Healing Rate

The short antibiotic strategy associated with DAIR or DA management allowed us to obtain an elevated SSI healing rate of 79.3%, and a rate of 69.0% when excluding material removal and hemipelvectomy. This result is as good as it gets in the literature [3,4]. In our practice, an aggressive DAIR is performed at the slightest suspicion of infection, with replacement of the mobile implants whenever possible. We associate an intravenous probabilistic antibiotic treatment, using the association piperacillin-tazobactam + linezolid [40,45], after performing bacteriological sampling secondarily tailored to the microorganisms detected intraoperatively (Supplementary Table S1). This probabilistic approach aims to provide early infection treatment so as to limit delayed or chronic infections of any material implanted for reconstruction [46]. It can be a multiple DAIR strategy, with iterative surgeries, as described in the literature in these conditions [3,20,24]. The total duration of the antibiotic treatment is usually up to 12 weeks for patients with implants [13,47]. In case of DAIR failure, different salvage strategies can be considered depending on the case (from implant removal in a curative approach to chronic fistula and suppressive therapy, or even external hemipelvectomy in critical situations).

#### 4.3.2. Factors Influencing the SSI Healing Rate

In terms of factors influencing the SSI healing rate, resistant bacteria have been identified less often in healed than in non-healed patients (50.0 vs. 77.8%), respectively. However, this was not confirmed in our multivariate logistic regression (*p* = 0.189). This could be due to a lack of power, but also to the difficulty with establishing a severity stratified risk for various resistances, leading to a simple yet objectionable dichotomy (no resistance vs. resistance). We also noted a tendency for a higher cure rate with implant retention in early infection (75.0 vs. 55.6%). This trend was also observed in our multivariate model, albeit without reaching significance (*p* = 0.217). In light of these results, we hypothesize that the low-resistance bacteriological profile that follows our short prophylaxis, combined with the early DA or DAIR management, may help increase the infection cure rate.

The most significant variable influencing SSI healing in our multivariate model remains the absence of an implant (*p* = 0.023), as SSIs are then clearly easier to manage. Thus, the current trend in our practice aims to limit material implantation after pelvic resections. In light of this, it has to be emphasized that half of our SSIs occurred without reconstruction initially, which may explain our high healing rate. It would be interesting to perform the same regression analysis on a larger cohort and to focus on patients with implants only, as this should increase the power of the model and allow further validation of early infection management and resistant profile variables.

#### 4.3.3. Implant Retention and DAIR

In case of reconstruction, implant removal was only necessary in 20% of cases, which is a low rate compared to what has been reported in the literature [3,48,49]. Other teams have reported lower SSI cure rates with implant retention. In particular, Angelini et al. described 46% implant removal, 9% external hemipelvectomy, and persistent chronic infection for 25% of their septic cases in the event of reconstruction [20]. Similarly, Sanders et al. reported 50% implant removal in a study focusing on pelvic reconstruction infection [3].

In addition to our short antibiotic regimen, these outcomes might be linked to the precocity of the SSI occurrence and management, as 75.9% occurred in the first month. The latter may be a good potential prognostic factor for infection healing with material retention, albeit without reaching significance in our multivariate model, possibly due to the small number of subjects and the predominant effect of material implantation (OR = 4.94 (CI 95%, 0.39–62.41), *p* = 0.217).

Nonetheless, in our series, a DAIR strategy, defined as early surgery with radical debridement exchange of removable components [24], allows implant retention in more than three cases out of four. We believe that a 24 h short antibiotic prophylaxis [17] may increase the rate of SSI healing, which may be due to early infection.

### 4.4. Study Limitations and Strengths

Our study’s limitations lie, first of all, in its design as a descriptive retrospective study, with heterogeneous inclusions inherent to the rare nature of the pathology. It should be noted that the ecological flora described in our work may differ from other areas, not necessarily only linked to the antibiotic prophylaxis, but also due to geographical variability and medical facility ecology. Secondly, our work was particularly focused on the microbiological flora and the resistance pattern related to a short antibiotic prophylaxis approach. Yet, it did not assess other factors that could be confounding biases in infection healing, such as negative-pressure wound therapy, coverage flaps, and adjuvant chemo or radiotherapy.

Our study’s strengths, on the other hand, lie in the detailed microbiological description and the resistance profile analysis of the bacteria implicated in the SSIs, which has not been described much to date for the resection of pelvic malignant tumors. Moreover, this work is the first to specifically study a short 24 h antibiotic prophylaxis regimen in sarcoma pelvic surgery.

Overall, considering the absence of the emergence of resistant bacteria, along with the SSI healing rate, our novel short 24 h antibiotic prophylaxis regimen in pelvic tumor resection warrants being considered in SSI prevention strategies.

## 5. Conclusions

As it does not present selective pressure favoring the emergence of multi-resistant bacteria, a short IV C1G of 2 g, with or without 1 g of IV nitroimidazole, could be a suitable prophylaxis regimen in specific pelvic sarcoma resection. Thus, the benefit of nitroimidazole adjunction in this strategy needs to be evaluated in a larger cohort. In this situation, within the context of a predominantly digestive flora and numerous polymicrobial presentations, SSI after tumoral pelvic resection can be managed with DA or DAIR procedures, thereby allowing a good SSI cure rate to be achieved with a low level of implant removal. Furthermore, our data suggest that the absence of material implantation is associated with infection healing. Early management and a low resistance profile may also have a positive effect on SSI healing and thus explain our results, but this needs to be confirmed in a larger cohort. As SSI is one of the most dreaded causes of reconstruction failure and morbidity during the first year, the simple prophylaxis protocol that we used here could be suitable for surgeons and infection teams to maximize implant retention, keeping in mind the patient functional prognosis, with a limited impact on bacterial ecology.

## Figures and Tables

**Table 1:** Demographic data for the SSI cohort.

		SSI Cohort (*n* = 29)
Characteristics		
	Age (years)	49.8 (±19.7)
	Male	22 (75.9%)
	BMI (kg/m^2^)	26.8 (±4.8)
	Follow-up (months)	56 (±38)
Localization		
	Iliac bone	20 (69.0%)
	Sacrum	9 (31.0%)
Histology		
	Chondrosarcoma	15 (51.7%)
	Osteosarcoma	5 (17.2%)
	Ewing	4 (13.8%)
	Chordoma	4 (13.8%)
	Others	1 (3.4%)
Co-morbidities		
	Diabetes	4 (13.8%)
	Active smoking	3 (10.3%)
	Immunosuppressive treatment	2 (6.9%)
	Inflammatory disease	1 (3.4%)
	Albumin (g/L)	28.0 (±8.1)
Scores		
	ASA	1.8 (±0.5)
	CCI	2.6 (±1.2)

SSI: Surgical site infection, BMI: Body Mass Index, ASA: American Anesthesiologist Association, CCI: Charlson Comorbidity Index.

**Table 2:** Resection surgery data for the SSI cohort.

		SSI Cohort (*n* = 29)
24 h Antibiotic prophylaxis		
	Monotherapy (1GC *)	21 (72.4%)
	Dual therapy (1GC * + nitroimidazole)	8 (27.6%)
Adjuvant treatment (global)		10 (34.5%)
	Chemotherapy	9 (31.0%)
	Radiotherapy	3 (10.3%)
Surgical margins		
	R0	16 (55.2%)
	R1	11 (34.5%)
	R2	2 (6.9%)
Cutting planning (global)		12 (41.4%)
	CT Navigation	1 (3.4%)
	PSI guide	11 (37.9%)
Reconstruction, implant		
	Prosthesis/Fixation	15 (51.7%)
Associated procedure		
	Combined visceral surgery	10 (34.5%)
	Pedicled flap	3 (10.3%)
Perioperative data		
	Surgical specimen volume (cm^3^)	750 (±725)
	Duration (min)	479 (±172)
	Blood loss (mL)	2690 (±1.786)
	Packed red blood cells	17.55 (±8.89)

* or vancomycin (×2) or clindamycin (×1) or cotrimoxazole (×1) in case of allergy; BMI: Body Mass Index, 1CG: First-generation cephalosporin, min: minutes, PSI: Patient specific instrument; Surgical specimen volume = (High × Length × Width)/2.

**Table 3:** Ecology and distribution of the microorganisms involved in the SSIs.

**Gram-Negative Bacilli (GNB): 26 (36.1%)**	***Escherichia coli***	**12 (16.7%)**
	*Proteus mirabilis*	5 (6.9%)
	*Enterobacter cloacae*	3 (4.2%)
	*Morganella morganii*	1 (1.4%)
	*Citrobacter koseri*	1 (1.4%)
	*Klebsiella oxytoca*	1 (1.4%)
	*Proteus vulgaris*	1 (1.4%)
	*Citrobacter freundii*	1 (1.4%)
	*Pseudomonas aeruginosa*	1 (1.4%)
Anaerobes: 10 (13.9%), except *Cutibacterium acnes*	*Bacteroides fragilis*	4 (5.6%)
	*Bacteroides ovatus*	1 (1.4%)
	*Bacteroides vulgatus*	1 (1.4%)
	*Bacteroides uniformis*	1 (1.4%)
	*Bacteroides* sp.	1 (1.4%)
	*Actinomyces turicensis*	1 (1.4%)
	*Prevotella* sp.	1 (1.4%)
*Enterococcus faecalis:*		15 (20.8%)
*Cutibacterium acnes:*		4 (5.6%)
Gram-Positive Cocci (GPC): 17 (23.6%), except *Enterococci*	*Staphylococcus aureus*	7 (9.7%)
	*Staphylococcus epidermidis*	6 (8.3%)
	Other coagulase-negative *staphylococci*	3 (4.2%)
	*Streptococcus mitis*	1 (1.4%)
**Total: 72**		

**Table 4:** Antibiotic prophylaxis, microorganism ecology, resistance, and healing rate.

Age	Gender	Antibiotic Prophylaxis	Microorganisms Involved in the SSI					Early Infection	Healing Status
57	F	1GC	*P. mirabilis*	*C. freundii*				Yes	Healed, material removal
62	M	1GC	*S. lugdunensis*					Yes	Healed
71	M	1GC	*E. coli* ^3^	*S. aureus*	*E. faecalis*	*P. vulgaris*		Yes	Healed
55	M	1GC	*E. coli* ^4^	*E. faecalis*	*Streptococcus mitis*			Yes	Healed
66	M	1GC	*E. coli* ^3^					No	Fistula, material retention
48	M	1GC	*E. cloacae*	*E. faecalis*				Yes	Healed
20	M	1GC	*S. aureus*					No	Healed
47	M	1GC	*E. coli* ^3^	*S. epidermidis*	*S. warnerii*			No	Healed, material removal
18	M	1GC	*E. faecalis*	*S. epidermidis* ^2^				No	External Hemipelvectomy
18	M	1GC	*E. coli* ^3^	*E. faecalis*	*Bacteroides* sp.	*Prevotella* sp.		Yes	Healed
51	M	1GC	*P. aeruginosa* ^6^	*S. epidermidis* ^1^	*C. acnes*			Yes	Healed
76	F	1GC	*P. mirabilis* ^3^	*E. faecalis*	*E. coli* ^4^	*B. fragilis*	*M. morganii* ^3^	Yes	Fistula, material removal
26	F	1GC	*E. coli*	*E. faecalis*				Yes	Healed
58	F	1GC	*E. coli* ^5^	*E. faecalis*				Yes	Healed
76	M	1GC	*S. aureus*	*C. koseri*	*C. acnes*			Yes	Healed
61	M	1GC	*S. epidermidis* ^2^	*E. faecalis*	*E. cloacae*			Yes	Fistula, material retention
56	M	1GC	*P. mirabilis*	*A. turicensis*	*B. ovatus* ^5^	*B. fragilis* ^5^		No	Fistula, no initial reconstruction
84	F	1GC	*S. aureus*					No	Healed
41	F	1GC + NI	*P. mirabilis*	*E. coli* ^3^	*E. faecalis*			No	Healed
64	M	1GC + NI	*S. capitis*	*C. acnes*				No	Healed
16	F	1GC + NI	*E. coli* ^3^	*E. faecalis*	*B. ovatus*	*B. fragilis*	*B. uniformis*	Yes	Healed
27	M	1GC + NI	*S. epidermidis* ^1^					Yes	Healed
70	M	1GC + NI	*E. coli* ^3^	*E. faecalis*				Yes	Healed
30	M	1GC + NI	*E. coli* ^3^	*E. faecalis*	*S. aureus*	*B. fragilis*		Yes	Healed
54	M	1GC + NI	*K. oxytoca*	*E. faecalis*	*S. aureus*			Yes	Healed
58	M	Clindamycin + NI	*P. mirabilis*					Yes	Healed
22	M	Cotrimoxazole	*E. faecalis*	*S. epidermidis* ^2^				Yes	Suppressive antibiotics
54	M	Vancomycin	*S. aureus*	*C. acnes*				Yes	Healed
59	M	Vancomycin	*E. cloacae*					Yes	Fistula, prosthesis retention

Early infection was defined as occurring in the first month following surgery. NI: nitroimidazole, SSI: Surgical site infection, 1GC: First-generation cephalosporin, Acquired antibiotic resistance: ^1^: Methicillin-resistant, ^2^: Multidrug-resistant (including methicillin), ^3^: Penicillinase, ^4^: Cephalosporinase, ^5^: Quinolone resistant, ^6^: Efflux MexAB6-prn.

**Table 5:** Multivariate logistic regression model for the infection healing probability.

Infection Healing Probability *		
	Model *p*-Value = 0.006	
Multivariate analysis	Coefficient (95.0% CI)	*p* Value
Early infection (<1 month)	4.94 (0.39–62.41)	*p* = 0.217
Material implantation	0.49 (0.04–0.66)	*p* = 0.023
Number of resistant bacteria†	0.43 (0.12–1.52)	*p* = 0.189

Each variable with a univariate regression analysis significance threshold of *p* < 0.15 was added to the multivariate analysis model. Univariate results: Material implantation (score = 7.22, degree of freedom (df):1, *p* = 0.007), Number of resistant bacteria† (score = 2.44, df:1, *p* = 0.119), Early infection (<1 month) (score = 2.42, df:1, *p* = 0.120), Initial 1GC 24-h monotherapy (score = 1.97, df:1, *p* = 0.160), ASA score (score = 2.38, df:1, *p* = 0.304), Albumin level† (score: 1.05, df:1, *p* = 0.307), Age† (score = 0.09, df:1, *p* = 0.763), Surgical specimen volume† (score = 0.80, df:1, *p* = 0.372), Body mass index † (score = 1.36, df:1, *p* = 0.244), Number of bacteria† (score = 0.21, df:1, *p* = 0.651), Associated radiation therapy (score = 0.78, df:1, *p* = 0.377); Diagnosis (score = 2.68, df:4, *p* = 0.613). * Excluding material removal and hemipelvectomy from healed patients, †: continuous variable.

## Data Availability

The datasets generated and/or analyzed during the current study are available from the corresponding author on reasonable request.

## Supplementary Materials

The following are available online at https://www.mdpi.com/article/10.3390/antibiotics10070768/s1, Table S1: Antibiotic prophylaxis, microorganism ecology, resistance, and targeted antibiotic regimen in patients with DAIR management in case of material implantation.
