# Supplementary material for: A Short-Course Antibiotic Prophylaxis Is Associated with Limited Antibiotic Resistance Emergence in Post-Operative Infection of Pelvic Primary Bone Tumor Resection
Source: Antibiotics (Basel). 2021 Jun 24;10(7):768. doi: 10.3390/antibiotics10070768 (PMC8300712; doi:10.3390/antibiotics10070768)
Supplement: Supplementary file 1 [file antibiotics-10-00768-s001.zip › antibiotics-1213248-supplementary.pdf]

antibiotics

Article

A Short-Course Antibiotic Prophylaxis Is Associated with Limited Antibiotic Resistance Emergence in Post-Operative Infection of Pelvic Primary Bone Tumor Resection

Yoann Varenne [1], Stéphane Corvec [2,3,4,5], Anne-Gaëlle Leroy [2,3,6], David Boutoille [3,7], Mỹ-Vân Nguyễn [1], Sophie Touchais [1,3], Pascale Bémer [2,3], Antoine Hamel [8,9], Denis Waast [1], Christophe Nich [1,10], François Gouin [1,11,†] and Vincent Crenn [1,3,10,*,†]

Supplementary Data:

Table S1. Antibiotic prophylaxis, microorganism ecology, resistance, and targeted antibiotic regimen in patients with DAIR management in case of material implantation.

Age	Gender	Antibiotic Prophylaxis	Microorganisms Involved in the SSI					Targeted Therapeutic Antibiotic Association Regimen after DAIR Management *		
16	F	1GC + NI	*E. coli*[3]	*E. faecalis*	*B. ovatus*	*B. fragilis*	*B. uniformis*	Piperacillin/tazobactam	Quinolone	
18	M	1GC	*E. faecalis*	*S. epidermidis*[2]				Daptomycin	Ertapenem	
22	M	Cotrimoxazole	*E. faecalis*	*S. epidermidis*[2]				Daptomycin	Rifampicin	
27	M	1GC + NI	*S. epidermidis*[1]					Rifampicin	Quinolone	
47	M	1GC	*E. coli*[3]	*S. epidermidis*	*S. warnerii*			Imipenem/cilastatin	Quinolone	
48	M	1GC	*E. cloacae*	*E. faecalis*				3GC	Cotrimoxazole	Quinolone
54	M	1GC + NI	*K. oxytoca*	*E. faecalis*	*S. aureus*			Rifampicin	Quinolone	Amoxicillin
57	F	1GC	*P. mirabilis*	*C. freundii*				Quinolone	3GC	
59	M	Vancomycin	*E. cloacae*					Quinolone	Cotrimoxazole	
62	M	1GC	*S. lugdunensis*					Quinolone	Clindamycin	
64	M	1GC + NI	*S. capitis*	*C. acnes*				Quinolone	Rifampicin	
66	M	1GC	*E. coli*[3]					3GC	Clindamycin	
71	M	1GC	*E. coli*[3]	*S. aureus*	*E. faecalis*	*P. vulgaris*		Amoxicillin/CA	Quinolone	
76	F	1GC	*P. mirabilis*[3]	*E. faecalis*	*E. coli*[4]	*B. fragilis*	*M. morganii*[3]	Amoxicillin/CA	Quinolone	
84	F	1GC	*S. aureus*					Clindamycin	Quinolone	

* Definitive treatment with infection board validation after bacteriological culture and antibiogram. 3-month duration. NI: nitroimidazole, CA: Clavulanic acid; SSI: Surgical site infection; DAIR: Debridement, antibiotics, and implant retention ; 1GC: First-generation cephalosporin, 3GC: Third-generation cephalosporin; Acquired antibiotic resistance: [1]: Methicillin-resistant, [2]: Multidrug-resistant (including methicillin), [3]: Penicillinase, [4]: Cephalosporinase, [5]: Quinolone resistant, [6]: Efflux MexAB6-prn.
